# 2-[(2,3,6,7-Tetra­hydro-1*H*,5*H*-benzo[*ij*]quinolizin-9-yl)methyl­ene]propane­dinitrile

**DOI:** 10.1107/S1600536809023678

**Published:** 2009-06-27

**Authors:** Min Liang, Hemant Yennawar, Mark Maroncelli

**Affiliations:** a Department of Chemistry, The Pennsylvania State University, 104 Chemistry Building, University Park, PA 16802, USA

## Abstract

The *π* system of  the title compound, known as julolidinemalononitrile, C_16_H_15_N_3_, is nearly planar, with a 3.5 (1)° twist between the aromatic and dicyano­vinyl groups. The bond lengths indicate significant zwitterionic character in the ground state.

## Related literature

For background to julolidinemalononitrile, see: Haidekker & Theodorakis (2007 ▶); Hooker & Torkelson (1995 ▶); Loutfy & Arnold (1982 ▶); Marder *et al.* (1993 ▶); Mennucci *et al.* (2009 ▶); Paul & Samanta (2008 ▶); Swalina & Maroncelli (2009 ▶). For related benzyl­idene malononitrile structure data see Wang *et al.* (2001 ▶); Anti­pin *et al.* (2003 ▶); van Bolhuis & Kiers (1978 ▶).
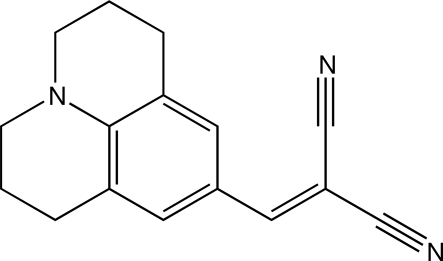


## Experimental

### 

#### Crystal data

C_16_H_15_N_3_
*M* *_r_* = 249.31
Monoclinic, 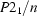

*a* = 4.9587 (9) Å
*b* = 15.614 (3) Å
*c* = 16.699 (3) Å
β = 91.609 (3)°
*V* = 1292.4 (4) Å^3^
*Z* = 4
Mo *K*α radiation
μ = 0.08 mm^−1^
*T* = 110 K
0.28 × 0.15 × 0.14 mm

#### Data collection

Bruker SMART CCD area-detector diffractometer
Absorption correction: multi-scan (*SADABS*; Bruker, 2003 ▶) *T*_min_ = 0.979, *T*_max_ = 0.989
7359 measured reflections
3158 independent reflections
2323 reflections with *I* > 2σ(*I*)
*R*_int_ = 0.045

#### Refinement

*R*[*F* ^2^ > 2σ(*F* ^2^)] = 0.070
*wR*(*F* ^2^) = 0.169
*S* = 1.06
3158 reflections
172 parameters
H-atom parameters not refined
Δρ_max_ = 0.47 e Å^−3^
Δρ_min_ = −0.28 e Å^−3^

### 

Data collection: *SMART* (Bruker, 2003 ▶); cell refinement: *SAINT* (Bruker, 2003 ▶); data reduction: *SAINT*; program(s) used to solve structure: *SHELXS97* (Sheldrick, 2008 ▶); program(s) used to refine structure: *SHELXL97* (Sheldrick, 2008 ▶); molecular graphics: *SHELXTL* (Sheldrick, 2008 ▶); software used to prepare material for publication: *SHELXTL*.

## Supplementary Material

Crystal structure: contains datablocks I, global. DOI: 10.1107/S1600536809023678/bt2965sup1.cif

Structure factors: contains datablocks I. DOI: 10.1107/S1600536809023678/bt2965Isup2.hkl

Additional supplementary materials:  crystallographic information; 3D view; checkCIF report

## Figures and Tables

**Fig. 1.**
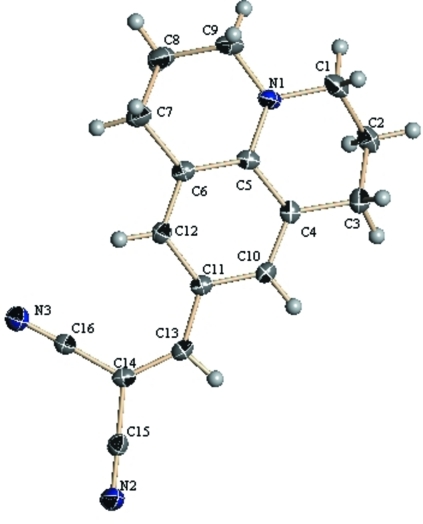
: Molecular structure of the title compound with displacement ellipsoids at the 50 % probability level.

**Fig. 2.**
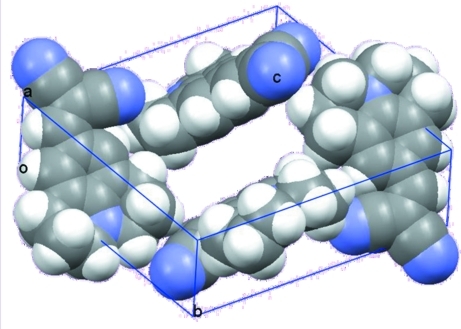
: Crystal packing of the title compound.

**Fig. 3.**
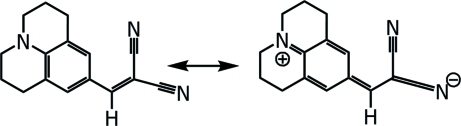
The formation of the title compound.

**Fig. 4.**
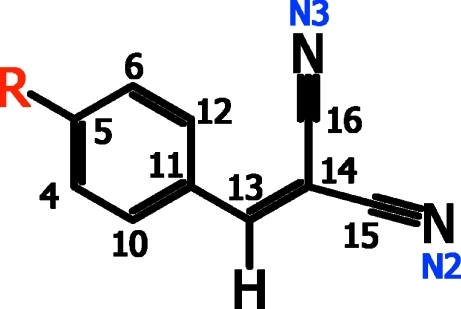
The molecular structure and the meaning of R relevant for Table 1.

## supplementary crystallographic information

### Comment

Julolidinemalononitrile, or JDMN for short (Fig. 1), has been extensively study for two distinct purposes. As a classic "push-pull" molecule with large hyperpolarizability, it has been used as a model system for understanding nonlinear optical properties of molecules (Mennucci *et al.*, 2009). In a completely different context, the environmental sensitivity of the emission yield of JDMN has also made it a popular probe for local fluidity in conventional solvents (Loutfy & Arnold, 1982), polymers (Hooker & Torkelson, 1995), ionic liquids (Paul & Samanta, 2008) and biological media (Haidekker & Theodorakis, 2007). In an effort to understand this environmental sensitivity, we have undertaken electronic structure calculations of JDMN and related molecules (Swalina & Maroncelli, 2009). To partially test the accuracy of these calculations we obtained crystal structure data on JDMN, which are reported here.

The crystal packing of JDMN consists of interleaved columns of slipped π-stacked molecules (Fig. 2). The molecular structure contains planar dicyanovinyl and aminobenzene portions, which are nearly coplanar with one another. The 3.0 (4)° torsion angle (C12—C11—C13—C14), as well as the wide C11—C13—C14 angle (131.6 (2)°; θ in Table 1), results from the unfavorable overlap between the hydrogen atom on C12 and N3 (2.724 (2) Å). The julolidine ring system of JDMN adopts a *syn* ("W") conformation in which the amino nitrogen atom is nearly *sp*^2^ hybridized (average 〈 CNC = 119.6 (2)°).

It is useful to view the ground and first excited state of JDMN as consisting of mixtures of the neutral and zwitterionic forms depicted in Scheme 2. Table 1 lists some structural parameters of JDMN useful in this context, together with data for several related molecules (see table 1). Ar1 and Ar2 are the average aromatic bond lengths that form the single (1) and double (2) bonds in the zwitterionic state; Δ_Ar_ is the difference between these bond lengths. As shown by the data in Table 1, JDMN and the closely related dimethylamino compound (DMN, *R* = N(CH_3_)_2_) exhibit non-negligible quinoidal character (Wang *et al.*, 2001), whereas the unsubstituted (*R* = H) and halogen-substituted analogues (Antipin *et al.*, 2003), represented here by *R* = F, do not. (For reference Δ_Ar_ = 0.14 Å in *p*-benzoquinone (van Bolhuis & Kiers, 1978)). Table 1 also lists bond lengths in the vinyl portion of the molecule, as well as Δ_pm_ the bond-length alternation in the polymethine chain. Δ_pm_ is small in both JDMN and DMN compared to the ideal polymethine value of 0.11 Å.(Marder *et al.*, 1993) In contrast, the values of Δ_pm_ in the H– and F-substituted analogues are much closer to the ideal. Both the difference in aromatic bond and vinyl bond lengths indicate substantial zwitterionic character in the ground state of the push-pull molecule JDMN (as well as in DMN).

### Experimental

JDMN was dissolved in a 1:1 (*v*/*v*) mixture of CH_2_Cl_2_ and ethanol. Orange block-shaped crystals of JDMN were grown by evaporating solvents slowly at room temperature.

### Refinement

H atom were positioned geometrically (C_aromatic_—H = 0.93 Å, C_methylene_—H = 0.97 Å)   and refined as riding with U_iso_(H) = 1.2U_eq_(C_aromatic_).

### Crystal data

**Table:**

C_16_H_15_N_3_	*F*(000) = 528
*M**_r_* = 249.31	*D*_x_ = 1.281 Mg m^−^^3^
Monoclinic, *P*2_1_/*n*	Mo *K*α radiation, λ = 0.71073 Å
*a* = 4.9587 (9) Å	Cell parameters from 1409 reflections
*b* = 15.614 (3) Å	θ = 2.8–26.9°
*c* = 16.699 (3) Å	µ = 0.08 mm^−^^1^
β = 91.609 (3)°	*T* = 110 K
*V* = 1292.4 (4) Å^3^	Block, orange
*Z* = 4	0.28 × 0.15 × 0.14 mm

### Data collection

**Table:**

Bruker SMAT CCD area-detector diffractometer	3158 independent reflections
Radiation source: fine-focus sealed tube	2323 reflections with *I* > 2σ(*I*)
graphite	*R*_int_ = 0.045
φ and ω scans	θ_max_ = 28.3°, θ_min_ = 1.8°
Absorption correction: multi-scan (*SADABS*; Bruker, 2003)	*h* = −6→6
*T*_min_ = 0.979, *T*_max_ = 0.989	*k* = −17→20
7359 measured reflections	*l* = −13→22

### Refinement

**Table:**

Refinement on *F*^2^	Primary atom site location: structure-invariant direct methods
Least-squares matrix: full	Secondary atom site location: difference Fourier map
*R*[*F*^2^ > 2σ(*F*^2^)] = 0.070	Hydrogen site location: inferred from neighbouring sites
*wR*(*F*^2^) = 0.169	H-atom parameters not refined
*S* = 1.06	*w* = 1/[σ^2^(*F*_o_^2^) + (0.0705*P*)^2^ + 0.5857*P*] where *P* = (*F*_o_^2^ + 2*F*_c_^2^)/3
3158 reflections	(Δ/σ)_max_ < 0.001
172 parameters	Δρ_max_ = 0.47 e Å^−^^3^
0 restraints	Δρ_min_ = −0.28 e Å^−^^3^

### Special details

**Table:**

Geometry. All e.s.d.'s (except the e.s.d. in the dihedral angle between two l.s. planes) are estimated using the full covariance matrix. The cell e.s.d.'s are taken into account individually in the estimation of e.s.d.'s in distances, angles and torsion angles; correlations between e.s.d.'s in cell parameters are only used when they are defined by crystal symmetry. An approximate (isotropic) treatment of cell e.s.d.'s is used for estimating e.s.d.'s involving l.s. planes.
Refinement. Refinement of *F*^2^ against ALL reflections. The weighted *R*-factor *wR* and goodness of fit *S* are based on *F*^2^, conventional *R*-factors *R* are based on *F*, with *F* set to zero for negative *F*^2^. The threshold expression of *F*^2^ > σ(*F*^2^) is used only for calculating *R*-factors(gt) *etc*. and is not relevant to the choice of reflections for refinement. *R*-factors based on *F*^2^ are statistically about twice as large as those based on *F*, and *R*- factors based on ALL data will be even larger.

### Fractional atomic coordinates and isotropic or equivalent isotropic displacement parameters (Å^2^)

**Table:**

	*x*	*y*	*z*	*U*_iso_*/*U*_eq_	
C1	0.2573 (4)	0.10806 (16)	0.50258 (14)	0.0280 (5)	
H1A	0.0851	0.1375	0.4999	0.034*	
H1B	0.2221	0.0469	0.5024	0.034*	
C2	0.4171 (5)	0.13118 (15)	0.43008 (14)	0.0283 (5)	
H2A	0.3123	0.1182	0.3817	0.034*	
H2B	0.5817	0.0977	0.4297	0.034*	
C3	0.4856 (4)	0.22589 (15)	0.43229 (13)	0.0254 (5)	
H3A	0.6008	0.2398	0.3881	0.030*	
H3B	0.3215	0.2594	0.4264	0.030*	
C4	0.6279 (4)	0.24797 (14)	0.51057 (12)	0.0193 (5)	
C5	0.5717 (4)	0.20016 (13)	0.58073 (13)	0.0188 (4)	
C6	0.6987 (4)	0.22407 (14)	0.65502 (12)	0.0197 (5)	
C7	0.6322 (5)	0.17692 (15)	0.73081 (14)	0.0278 (5)	
H7A	0.4936	0.2080	0.7586	0.033*	
H7B	0.7916	0.1744	0.7658	0.033*	
C8	0.5344 (5)	0.08689 (16)	0.71287 (15)	0.0318 (6)	
H8A	0.6833	0.0523	0.6947	0.038*	
H8B	0.4673	0.0611	0.7612	0.038*	
C9	0.3142 (4)	0.08886 (15)	0.64948 (14)	0.0266 (5)	
H9A	0.2593	0.0307	0.6367	0.032*	
H9B	0.1592	0.1188	0.6699	0.032*	
C10	0.8160 (4)	0.31257 (14)	0.51565 (12)	0.0191 (4)	
H10	0.8534	0.3430	0.4694	0.023*	
C11	0.9550 (4)	0.33494 (14)	0.58745 (12)	0.0186 (4)	
C12	0.8864 (4)	0.28912 (14)	0.65663 (12)	0.0200 (5)	
H12	0.9710	0.3033	0.7052	0.024*	
C13	1.1540 (4)	0.40053 (14)	0.58368 (12)	0.0204 (5)	
H13	1.1667	0.4259	0.5335	0.024*	
C14	1.3297 (4)	0.43292 (14)	0.64049 (12)	0.0194 (4)	
C15	1.5117 (4)	0.50020 (14)	0.62010 (13)	0.0221 (5)	
C16	1.3544 (4)	0.40483 (14)	0.72205 (13)	0.0198 (4)	
N1	0.4020 (3)	0.13133 (12)	0.57683 (11)	0.0233 (4)	
N2	1.6579 (4)	0.55454 (13)	0.60473 (11)	0.0301 (5)	
N3	1.3789 (4)	0.38375 (13)	0.78791 (12)	0.0290 (5)	

### Atomic displacement parameters (Å^2^)

**Table:**

	*U*^11^	*U*^22^	*U*^33^	*U*^12^	*U*^13^	*U*^23^
C1	0.0236 (11)	0.0236 (13)	0.0364 (13)	−0.0022 (9)	−0.0050 (9)	−0.0035 (10)
C2	0.0272 (11)	0.0275 (13)	0.0299 (12)	0.0010 (10)	−0.0054 (9)	−0.0076 (10)
C3	0.0272 (11)	0.0261 (13)	0.0226 (11)	0.0027 (9)	−0.0032 (9)	−0.0011 (9)
C4	0.0194 (10)	0.0182 (11)	0.0204 (10)	0.0055 (8)	0.0009 (8)	−0.0025 (8)
C5	0.0158 (9)	0.0156 (11)	0.0252 (11)	0.0029 (8)	0.0047 (8)	−0.0021 (8)
C6	0.0209 (10)	0.0160 (11)	0.0225 (11)	0.0036 (8)	0.0047 (8)	0.0005 (8)
C7	0.0338 (12)	0.0249 (13)	0.0251 (12)	−0.0042 (10)	0.0063 (9)	0.0022 (10)
C8	0.0331 (13)	0.0279 (14)	0.0347 (13)	−0.0044 (10)	0.0043 (10)	0.0080 (11)
C9	0.0240 (11)	0.0187 (12)	0.0375 (13)	−0.0016 (9)	0.0061 (9)	0.0017 (10)
C10	0.0209 (10)	0.0188 (11)	0.0177 (10)	0.0026 (8)	0.0025 (8)	0.0019 (8)
C11	0.0191 (10)	0.0160 (11)	0.0209 (10)	0.0029 (8)	0.0039 (8)	0.0001 (8)
C12	0.0229 (10)	0.0199 (12)	0.0174 (10)	0.0026 (8)	0.0014 (8)	−0.0007 (8)
C13	0.0228 (10)	0.0197 (12)	0.0189 (10)	0.0020 (8)	0.0043 (8)	0.0027 (9)
C14	0.0203 (10)	0.0161 (11)	0.0220 (10)	0.0009 (8)	0.0059 (8)	−0.0008 (9)
C15	0.0266 (11)	0.0212 (12)	0.0183 (10)	0.0009 (9)	0.0003 (8)	−0.0002 (9)
C16	0.0189 (10)	0.0184 (11)	0.0221 (11)	0.0001 (8)	0.0025 (8)	−0.0023 (9)
N1	0.0210 (9)	0.0204 (10)	0.0284 (10)	−0.0037 (7)	0.0001 (7)	0.0008 (8)
N2	0.0379 (11)	0.0271 (12)	0.0252 (10)	−0.0081 (9)	0.0004 (8)	0.0005 (9)
N3	0.0302 (10)	0.0312 (12)	0.0257 (11)	−0.0018 (8)	0.0009 (8)	−0.0005 (9)

### Geometric parameters (Å, °)

**Table:**

C1—N1	1.461 (3)	C7—H7B	0.9700
C1—C2	1.509 (3)	C8—C9	1.500 (3)
C1—H1A	0.9700	C8—H8A	0.9700
C1—H1B	0.9700	C8—H8B	0.9700
C2—C3	1.518 (3)	C9—N1	1.460 (3)
C2—H2A	0.9700	C9—H9A	0.9700
C2—H2B	0.9700	C9—H9B	0.9700
C3—C4	1.508 (3)	C10—C11	1.410 (3)
C3—H3A	0.9700	C10—H10	0.9300
C3—H3B	0.9700	C11—C12	1.409 (3)
C4—C10	1.375 (3)	C11—C13	1.425 (3)
C4—C5	1.423 (3)	C12—H12	0.9300
C5—N1	1.365 (3)	C13—C14	1.367 (3)
C5—C6	1.425 (3)	C13—H13	0.9300
C6—C12	1.377 (3)	C14—C15	1.432 (3)
C6—C7	1.509 (3)	C14—C16	1.433 (3)
C7—C8	1.514 (3)	C15—N2	1.150 (3)
C7—H7A	0.9700	C16—N3	1.151 (3)
			
N1—C1—C2	111.41 (18)	C9—C8—C7	110.1 (2)
N1—C1—H1A	109.3	C9—C8—H8A	109.6
C2—C1—H1A	109.3	C7—C8—H8A	109.6
N1—C1—H1B	109.3	C9—C8—H8B	109.6
C2—C1—H1B	109.3	C7—C8—H8B	109.6
H1A—C1—H1B	108.0	H8A—C8—H8B	108.2
C1—C2—C3	109.62 (19)	N1—C9—C8	111.58 (18)
C1—C2—H2A	109.7	N1—C9—H9A	109.3
C3—C2—H2A	109.7	C8—C9—H9A	109.3
C1—C2—H2B	109.7	N1—C9—H9B	109.3
C3—C2—H2B	109.7	C8—C9—H9B	109.3
H2A—C2—H2B	108.2	H9A—C9—H9B	108.0
C4—C3—C2	110.05 (18)	C4—C10—C11	123.28 (19)
C4—C3—H3A	109.7	C4—C10—H10	118.4
C2—C3—H3A	109.7	C11—C10—H10	118.4
C4—C3—H3B	109.7	C12—C11—C10	116.62 (19)
C2—C3—H3B	109.7	C12—C11—C13	125.78 (19)
H3A—C3—H3B	108.2	C10—C11—C13	117.59 (19)
C10—C4—C5	118.83 (19)	C6—C12—C11	122.5 (2)
C10—C4—C3	121.43 (19)	C6—C12—H12	118.8
C5—C4—C3	119.73 (19)	C11—C12—H12	118.8
N1—C5—C4	120.55 (19)	C14—C13—C11	131.6 (2)
N1—C5—C6	120.29 (19)	C14—C13—H13	114.2
C4—C5—C6	119.15 (19)	C11—C13—H13	114.2
C12—C6—C5	119.47 (19)	C13—C14—C15	120.00 (19)
C12—C6—C7	120.5 (2)	C13—C14—C16	125.6 (2)
C5—C6—C7	120.06 (19)	C15—C14—C16	114.40 (18)
C6—C7—C8	111.31 (19)	N2—C15—C14	179.1 (2)
C6—C7—H7A	109.4	N3—C16—C14	178.3 (2)
C8—C7—H7A	109.4	C5—N1—C9	121.04 (19)
C6—C7—H7B	109.4	C5—N1—C1	121.57 (19)
C8—C7—H7B	109.4	C9—N1—C1	116.21 (18)
H7A—C7—H7B	108.0		
			
N1—C1—C2—C3	−56.0 (2)	C5—C6—C12—C11	1.4 (3)
C1—C2—C3—C4	54.6 (2)	C7—C6—C12—C11	180.00 (19)
C2—C3—C4—C10	149.2 (2)	C10—C11—C12—C6	1.6 (3)
C2—C3—C4—C5	−29.5 (3)	C13—C11—C12—C6	−177.4 (2)
C10—C4—C5—N1	−174.91 (18)	C12—C11—C13—C14	3.0 (4)
C3—C4—C5—N1	3.9 (3)	C10—C11—C13—C14	−176.0 (2)
C10—C4—C5—C6	4.0 (3)	C11—C13—C14—C15	−179.9 (2)
C3—C4—C5—C6	−177.27 (18)	C11—C13—C14—C16	0.7 (4)
N1—C5—C6—C12	174.60 (18)	C13—C14—C15—N2	154 (17)
C4—C5—C6—C12	−4.3 (3)	C16—C14—C15—N2	−26 (17)
N1—C5—C6—C7	−4.0 (3)	C13—C14—C16—N3	−179 (100)
C4—C5—C6—C7	177.16 (18)	C15—C14—C16—N3	2(9)
C12—C6—C7—C8	−153.2 (2)	C4—C5—N1—C9	−171.71 (18)
C5—C6—C7—C8	25.4 (3)	C6—C5—N1—C9	9.4 (3)
C6—C7—C8—C9	−50.6 (3)	C4—C5—N1—C1	−4.6 (3)
C7—C8—C9—N1	56.3 (3)	C6—C5—N1—C1	176.57 (19)
C5—C4—C10—C11	−0.8 (3)	C8—C9—N1—C5	−36.2 (3)
C3—C4—C10—C11	−179.57 (19)	C8—C9—N1—C1	156.0 (2)
C4—C10—C11—C12	−2.0 (3)	C2—C1—N1—C5	31.3 (3)
C4—C10—C11—C13	177.13 (19)	C2—C1—N1—C9	−160.98 (19)

### Table 1 Table 1. Selected parameters (Å, °) and comparison to related molecules

**Table:**

Molecule	R—C5	Ar1	Ar2	Δ_Ar_	C11—C13	C13—C14	C—CN	Δ_pm_	C≡N	θ	τ	
JDMN*^a^*	1.365	1.417	1.376	0.04	1.425	1.367	1.433	0.06	1.151	132	3	
N(CH_3_)_2_*^b^*	1.359	1.410	1.366	0.04	1.423	1.367	1.431	0.06	1.142	132	7	
H*^c^*		1.387	1.379	0.01	1.450	1.350	1.440	0.09	1.145	131	10	
F*^c^*	1.356	1.377	1.380	0.00	1.454	1.341	1.437	0.10	1.133	132	5	

Notes: Ar1 = average {C10—C11, C11—C12, C4—C5, C5—C6}; Ar2 =  average {C4—C10, C6—C12};  Δ = Ar1–Ar2 C—CN = average{C14—C15, C14—C16};  Δpm = average{C11—C13, C14—C15, C14—C16} - (C13—C14); θ = the C11—C13—C14 angle; τ = the C12—C11—C13—C14 torsion angle. Notes: (*a*) This work; (*b*) Wang *et al.* (2001); (*c*) Antipin *et al.* (2003).
